# A rare cause of small bowel obstruction caused by duodenum-derived aggressive fibromatosis with β-catenin T41A mutation: A case analysis

**DOI:** 10.1097/MD.0000000000038984

**Published:** 2024-07-12

**Authors:** Dewen Zhao, Xinguang Wang

**Affiliations:** a Department of General surgery, Traditional Chinese Medicine Hospital of Jingtai County, Baiyin, Gansu, China.

**Keywords:** aggressive fibromatosis, case report, duodenum, small bowel obstruction, treatment

## Abstract

**Rationale:**

Aggressive fibromatosis (AF) is a fibroblastic/myofibroblastic tumor known for its locally aggressive properties. Intra-abdominal AF primarily occurs in the small intestine mesentery, ileocolic mesocolon, omentum, retroperitoneum, and pelvis, and rarely originates from the intestinal wall. Here, we report a rare case of small bowel obstruction caused by duodenum-derived AF with β-catenin (CTNNB1) T41A mutation.

**Patient concerns:**

A 35-year-old male had a 4-month history of abdominal pain, nausea, and vomiting, which gradually worsened over time.

**Diagnoses:**

Based on the results of CT examination, histopathology and Sanger sequencing, the patient was diagnosed with small bowel obstruction caused by duodenum-derived AF.

**Interventions:**

Due to the extensive adhesion between the tumor and surrounding tissue, it is extremely challenging to completely remove the tumor through surgical resection with negative margins in this case. In order not to damage the function of surrounding vital organs, gastrojejunostomy was performed to relieve the symptoms of small bowel obstruction.

**Outcomes:**

The patient experienced a successful recovery. It is important to note that this patient is still at risk of local recurrence and requires regular follow-up.

**Lessons:**

The best treatment should be taken based on the individual patient to relieve symptoms and improve quality of life. Moreover, histopathology plays a crucial role in diagnosing and differentiating duodenum-derived AF. The detection of mutations in exon 3 of the CTNNB1 has become strong evidence for diagnosing duodenum-derived AF.

## 1. Introduction

Aggressive fibromatosis (AF) is a fibroblastic/myofibroblastic tumor known for its locally aggressive properties. It is characterized by the production of abundant collagen fibers, a tendency for local recurrence, and a lack of metastasis.^[1]^ Invasive fibromatosis can be categorized into 3 types based on its location: extra-abdominal (50–60%), abdominal-wall (25%), and intra-abdominal fibromatosis (8%). The most common type is extra-abdominal AF, which usually occurs in the shoulders, followed by the chest wall, back, bones, and head and neck. Abdominal-wall AF typically develops in the rectus abdominis or internal oblique fascia. Intra-abdominal AF primarily occurs in the small intestine mesentery, ileocolic mesocolon, omentum, retroperitoneum, pelvis, and small intestine. There is currently limited data on the incidence of AF in the small intestine. The overall incidence rate of intra-abdominal AF is only 8%,^[2]^ and occurrences in the small intestine are even rarer. While case reports have documented AF in the jejunum, ileum, and small intestine mesentery,^[3–5]^ there are no reports of it occurring in the duodenum. Here, we report a rare case of small bowel obstruction caused by duodenum-derived AF with CTNNB1 T41A mutation.

## 2. Case report

**Figure 1.**
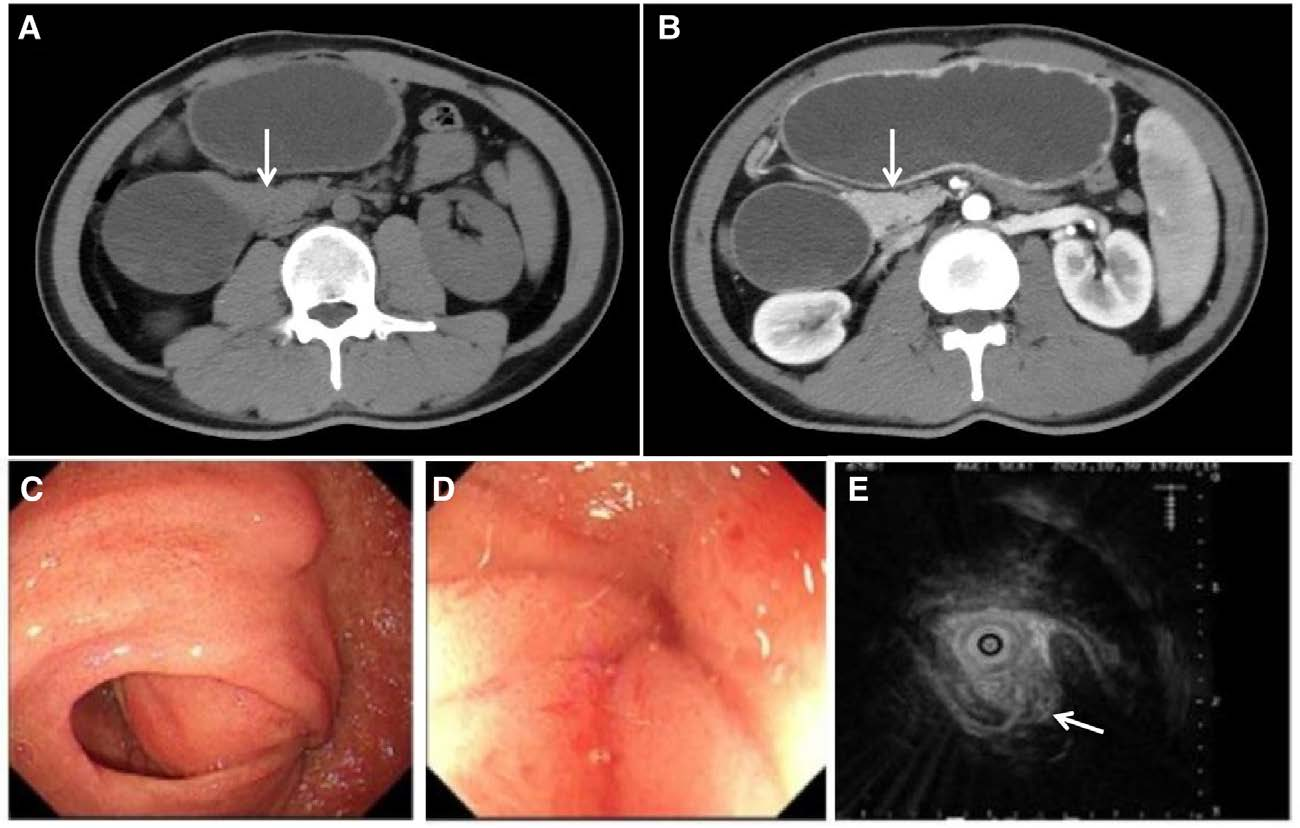
(A, B) CT with/without (i.v.) contrast (administration) revealed a large soft tissue mass in the descending part of duodenum, resulting in proximal intestinal dilation and gastric retention. (C) Gastroscopy showed a significant bulge in the descending segment of the duodenum, (D) narrowing the lumen and obstructing the passage of the endoscope. (E) Ultrasound shows a hypoechoic mass outside the descending duodenal wall, with no detectable boundaries.

**Figure 2.**
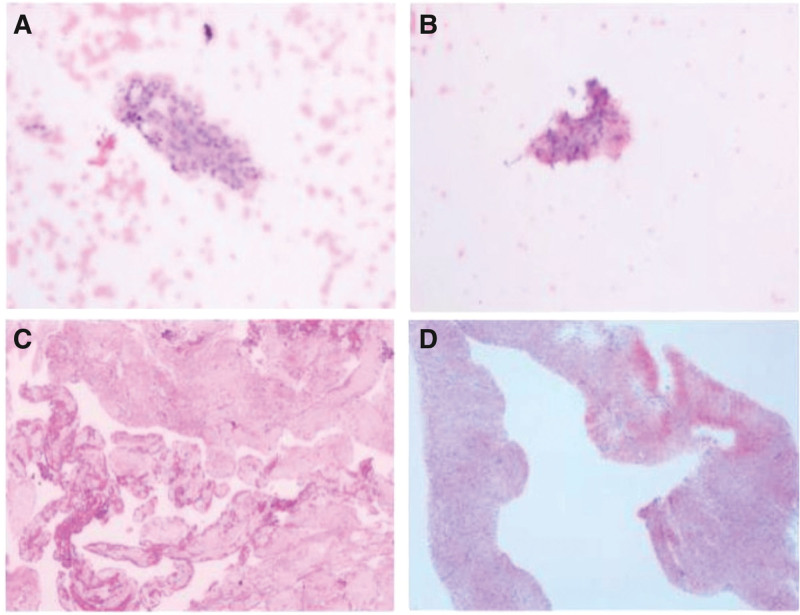
(A, B) Brush biopsy of the descending duodenum revealed the presence of a few round/short spindle cells. (C, D) CT-guided puncture biopsy of the abdominal mass was performed, and the pathological results indicated spindle cell hyperplasia.

**Figure 3.**
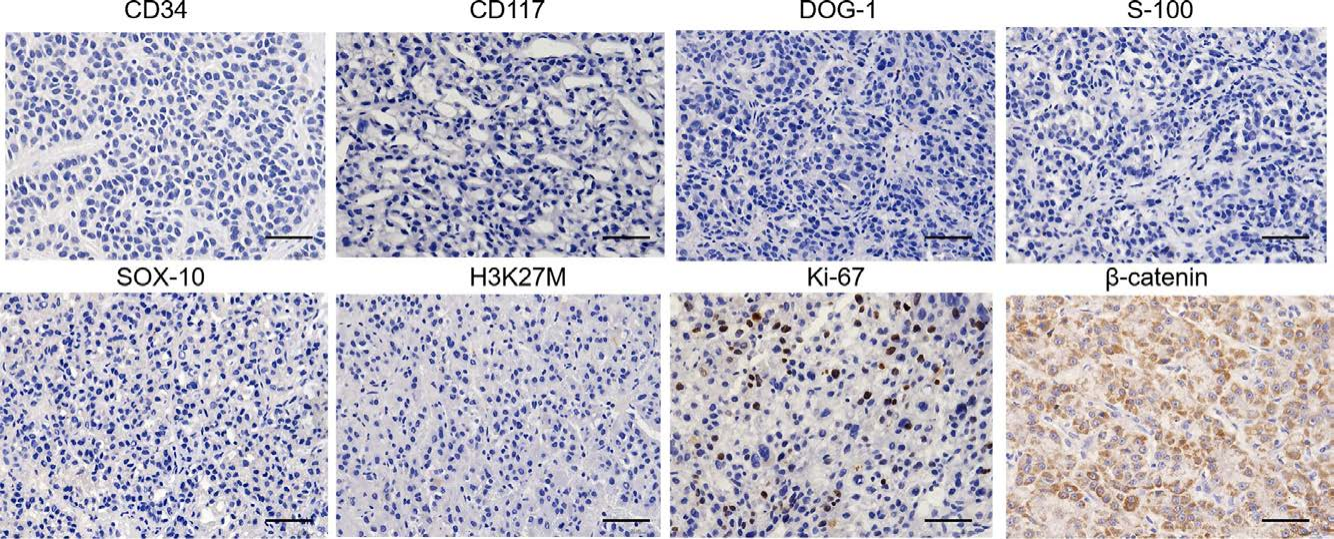
Immunohistochemical experiments showed CD34 (−), CD117 (−), DOG-1 (−), S-100 (−), SOX-10 (−), H3K27M (−), Ki-67 (<35%+), β-catenin (+) (immunohistochemical staining, 10×).

A 35-year-old male presented with abdominal pain that occurred 4 months ago. The pain was characterized by intermittent distension and localized discomfort, without radiation or sharp, stabbing sensations. The patient also experienced nausea and vomiting 3 times, with the vomited material consisting of food and gastric juice, approximately 80 mL per episode. There were no accompanying symptoms of fever or diarrhea. On examination, the patient had a heart rate of 110 beats/min and a blood pressure of 110/94 mm Hg. Laboratory test results revealed a white blood cell count of 3.29 × 10^9^/L, hemoglobin level of 137g/L, and platelet count of 162 × 10^9^/L. Serum tumor markers, including carcinoembryonic antigen, alpha-fetoprotein, and tumor-associated antigen 125, were within normal range. CT with/without (i.v.) contrast (administration) revealed a large soft tissue mass in the descending part of duodenum, resulting in proximal intestinal dilation and gastric retention (Fig. 1A and B). Gastroscopy showed a significant bulge in the descending segment of the duodenum (Fig. 1C), narrowing the lumen and obstructing the passage of the endoscope (Fig. 1D). Ultrasound shows a hypoechoic mass outside the descending duodenal wall, with no detectable boundaries (Fig. 1E). The duodenal mucosa at the bulge area is hyperemia without any obvious erosion or ulceration. Brush biopsy of the descending duodenum revealed the presence of a few round/short spindle cells (Fig. 2A and B). Subsequently, a CT-guided puncture biopsy of the abdominal mass was performed, and the pathological results indicated spindle cell hyperplasia (Fig. 2C and D). Initially, a gastrointestinal stromal tumor (GIST) was suspected. GIST typically show positive expression for CD34, CD117, and DOG-1, while duodenum-derived AF is negative for these markers.^[3]^ In this case, the postoperative pathology showed that the tumor cells were negative for CD34, CD117, DOG-1, S-100, SOX-10, and H3K27M, which confirmed the diagnosis of duodenum-derived AF (Fig. 3). Additionally, strong positive expression of β-catenin (CTNNB1) was observed (Fig. 3), further supporting the diagnosis of duodenum-derived AF.^[4]^ These images were fully discussed and confirmed with pathologists. Moreover, recent studies have shown that CTNNB1 mutations can serve as compelling evidence for diagnosing AF.^[5]^ In this case, Sanger sequencing detected the T41A mutation in exon 3 of the CTNNB1 gene, further supporting the diagnosis of duodenum-derived AF. Based on the histopathology, immunohistochemistry, and molecular pathology results, the patient was diagnosed with duodenum-derived AF. To alleviate symptoms of small bowel obstruction, the patient underwent gastrojejunostomy surgery and experienced a successful recovery. It is important to note that this patient is still at risk of local recurrence and requires regular follow-up.

## 3. Discussion

Small intestine tumors mainly include stromal tumor, lipoma, fibroma, neurofibroma, adenocarcinoma, malignant lymphoma, etc.^[6]^ AF is very rare in duodenum tumors. It is important to differentiate duodenum-derived AF from GIST. GIST typically show positive expression for CD34, CD117, and DOG-1, while duodenum-derived AF is negative for these markers.^[7]^ In this case, the postoperative pathology showed that the tumor cells were negative for CD34, CD117, DOG-1, S-100, and Desmin, which confirmed the diagnosis of duodenum-derived AF. Additionally, strong positive expression of CTNNB1 was observed, further supporting the diagnosis of duodenum-derived AF.^[8]^ The increase of CTNNB1 is a driving and progression factor for AF. Moreover, recent studies have shown that CTNNB1 mutations can serve as compelling evidence for diagnosing AF.^[9]^ The most common gene mutations of CTNNB1 in AF are found at the T41A, S45F, and S45P sites of the third exon. Studies have shown that the N-terminal domain of CTNNB1 contains S45 and T41 residues, which regulate the stabilization of CTNNB1.^[10]^ In addition, a study has shown that T41A or S45F mutations can weaken immunotherapy by affecting the infiltration of T cells and tumor-associated macrophages in the tumor microenvironment of AF.^[10]^ In this case, the T41A mutation in exon 3 of the CTNNB1 was detected through Sanger sequencing, further confirming the diagnosis of duodenum-derived AF.

What makes this case interesting is that the lesion is located in the descending duodenum, which has a very low incidence. Currently, there is only one similar report, where the site of lesion was located in the horizontal segment of the duodenum. However, the specific type of CTNNB1 exon 3 mutation remains unknown.^[11]^ Due to extensive adhesion between the tumor and surrounding tissue, complete removal of the tumor tissue through surgery was extremely challenging in this case. To alleviate the patient’s intestinal obstruction symptoms, a gastrojejunostomy was performed. Despite the effectiveness of surgical treatment, the local recurrence rate of the disease is as high as 24% to 77%.^[12]^ The patient underwent follow-up every 3 months postsurgery, which included imaging studies and tumor marker testing. At the most recent follow-up visit (12 months postsurgery), the patient showed no signs of recurrence and was in good health. Due to the high risk of recurrence associated with this disease, regular follow-up will be maintained to promptly detect and treat any potential recurrence.

## 4. Conclusion

Histopathology and immunohistology markers play a crucial role in diagnosing and differentiating duodenum-derived AF. The detection of mutations in exon 3 of the CTNNB1 through Sanger sequencing has become strong evidence for diagnosing duodenum-derived AF.

## Author contributions

**Conceptualization:** Xinguang Wang, Dewen Zhao.

**Data curation:** Xinguang Wang.

**Formal analysis:** Xinguang Wang.

**Writing – original draft:** Xinguang Wang.

**Methodology:** Dewen Zhao.

**Validation:** Dewen Zhao.

**Visualization:** Dewen Zhao.

**Writing – review & editing:** Dewen Zhao.

## References

[1] Gronchi A Jones RL. Treatment of desmoid tumors in 2019. JAMA Oncol. 2019;5:567–8. 30703188 10.1001/jamaoncol.2018.6449

[2] Okada T Hirata K Tanaka H. A case of intra-abdominal desmoid tumors occurring four years after open radical prostatectomy. Gan To Kagaku Ryoho. 2020;47:1836–8. 33468845

[3] Kamiya N Hashimoto I Otani K. A case of desmoid fibromatosis of the small intestinal mesentery institutions. Gan To Kagaku Ryoho. 2023;50:1104–6. 38035845

[4] Eswaravaka SK Deshpande SH Chiranjeev R. Intra-abdominal small intestinal desmoid tumour mimicking GIST. BMJ Case Rep. 2021;14:e237032. 10.1136/bcr-2020-237032 PMC7903087 33619130

[5] Friedberg S Hanan I Hyman N. Ileal desmoid tumor in Ileal Crohn’s: coincidence or connection? Dig Dis Sci. 2023;68:1071–3. 35713837 10.1007/s10620-022-07583-9

[6] Vlachou E Koffas A Toumpanakis C. Updates in the diagnosis and management of small-bowel tumors. Best Pract Res Clin Gastroenterol. 2023;64–65:101860. 10.1016/j.bpg.2023.101860 37652650

[7] Ji H Zhu W Zhao B. A giant mesenteric fibromatosis involving the muscular layer of the colon wall: a case report. Medicine (Baltimore). 2019;98:e14015. 30608449 10.1097/MD.0000000000014015 PMC6344171

[8] Prendergast K Kryeziu S Crago AM. The evolving management of desmoid fibromatosis. Surg Clin North Am. 2022;102:667–77. 35952695 10.1016/j.suc.2022.05.005

[9] Crago AM Chmielecki J Rosenberg M. Near universal detection of alterations in CTNNB1 and Wnt pathway regulators in desmoid-type fibromatosis by whole-exome sequencing and genomic analysis. Genes Chromosomes Cancer. 2015;54:606–15. 26171757 10.1002/gcc.22272 PMC4548882

[10] Colombo C Belfiore A Paielli N. beta-Catenin in desmoid-type fibromatosis: deep insights into the role of T41A and S45F mutations on protein structure and gene expression. Mol Oncol. 2017;11:1495–507. 28627792 10.1002/1878-0261.12101 PMC5664003

[11] Akbulut S Yilmaz M Alan S. Coexistence of duodenum derived aggressive fibromatosis and paraduodenal hydatid cyst: a case report and review of literature. World J Gastrointest Surg. 2018;10:90–4. 30510634 10.4240/wjgs.v10.i8.90 PMC6259024

[12] Vallabha T Sindgikar V Baloorkar R. Desmoid infilterating ileum, a rare complication. Indian J Surg. 2013;75(Suppl 1):192–4. 10.1007/s12262-012-0564-y PMC3693370 24426561

